# Deliver us from evil

**DOI:** 10.1017/S1478951525101600

**Published:** 2026-01-13

**Authors:** Ethan Schimmoeller

**Affiliations:** Hospice and Palliative Medicine, Riverside Methodist Hospital, Columbus, OH, USA

I often describe my work as a palliative care physician as pastoral medicine, underscoring the way that caring for seriously ill patients necessitates blurring the distinctions we’ve come to expect in healthcare. Of course, the interdisciplinary aspect of care is well acknowledged in the field. Yet, while palliative physicians have become part-social worker, part-psychologist, and part-pharmacist, how comfortable are we as part-chaplain? I do not advocate for physicians to provide spiritual counsel to patients routinely, but the doctor-as-chaplain is not discussed often enough. As a new attending physician out of training, a patient asked me to be his chaplain; in fact, he specifically requested we not call a chaplain, preferring that I attend to his spiritual needs. This is obviously not something I was taught in medical school!

Let’s call the patient Robert. I was consulted on Robert’s care to help clarify his goals of care with a likely new diagnosis of metastatic prostate cancer. One of our social workers and I sat with him as he shared his octogenarian life review in some detail. It became apparent that Robert’s soul was guilt-stricken as he returned to this theme repeatedly throughout our conversation. He identified himself as a Christian from a mainline Protestant denomination. Over and over again, he asked me: “Will God accept me?” Ordinarily, Christian moral theology views guilt as a symptom of sin or wrongdoing; it is a healthy conscience doing what it ought to do to provoke a person to seek forgiveness and reform their life. His confessed shortcomings, though, appeared quite ordinary and even banal, or perhaps scrupulous. In this case, Robert was tempted to despair.

Despair is the sin opposed to the theological virtue of hope, the virtue by which Christians confidently look forward to the kingdom of heaven, trusting in the promises of Jesus, hoping for the fulfillment of what we cannot yet see. Hope is “a sure and steadfast anchor of the soul” (Hebrews 6:19) stabilizing it to live faithfully in times of trial. For Robert his guilt prevented him from prayer, reading the Bible, or allowing a chaplain in the room. Instead of a means to forgiveness and mercy, it was an obstacle, and a serious one at that. Despair obstructs the primary task of a Christian in preparing to meet God upon death, and can shut off the soul from the possibility of forgiveness and thus salvation by falsely believing oneself to be unlovable or “too far gone.” It is a sin against the truth. In the Biblical new testament, despair is often identified with the “sin against the Holy Spirit” (Matthew 12:31) and Judas’s suicide after betraying Christ to his crucifixion (Matthew 27:5).

As a pastoral matter, how can one respond to Robert’s question authentically without falling into superficial spiritual reassurance? A recent volume, *The Art of Dying: A New Annotated Translation*, from physician and Dominican friar Columba Thomas provides a nice starting point, though some background is in needed first (2021). The “art of dying” (*Ars Moriendi* in Latin) was a popular 15th century Roman Catholic text intended to help Christians spiritually prepare for death, a final chance for repentance on the deathbed before the soul is judged after departing the body. As Thomas discusses, medieval piety featured robust belief both in realities after death and the spiritual power of the Church’s sacraments (Cf. Aries 1981). In fact, the text was intended not only for the sick and their caregivers but also for the healthy. The art of dying was a mirror to the art of living, not unlike our observation today that people tend to die in a manner resonant with how they lived. There was also a very practical pastoral problem facing 15th century Europe: countless priests died from the plague through their pastoral work leading to a serious clergy shortage to minister to the sick and dying faithful. So, the medieval text was a kind of spiritual “self-help” book for Christians without access to a priest. Recent authors have drawn inspiration from the medieval text to engage a broader Christian or secular audience (Dugdale 2020; Verhey 2011).

The *art of dying* text from Thomas reproduces much of the “abridged” medieval text with laudable annotation. The core of the text presents five temptations from wicked demonic spirits and five inspirations from holy angelic spirits to counter them centering upon the theological virtues and seven deadly sins. First, the temptation against faith and inspiration to faith; second, the temptation to despair and inspiration against despair (hope); third, the temptation to impatience and inspiration against impatience (charity, love); fourth, the temptation to vainglory (pride) and inspiration against vainglory (humility); fifth, the temptation to avarice (greed) and inspiration against avarice (detachment, generosity). These five couplets are accompanied by woodcut images that the faithful, especially illiterate believers, could “read” to communicate the same pastoral advice. Following these, Thomas’s text includes a selection of traditional prayers and devotional practices, notably the prayers for commendation of the soul, the rosary, and examination of conscience to prepare for sacramental confession of sins (Cf. Lampard 2005).

So what does the *art of dying* advise regarding despair? To start, the first sentence of the text appreciates the serious trial my patient, Robert, was encountering. Contra Aristotle who famously said, “of all terrible things, the death of the body is the most terrible,” Robert would certainly agree the soul is much more valuable than the body. “All the world combined does not equal the value of a single soul,” and, indeed, “the devil also knows how precious the soul is…it is of utmost importance for the dying person to tend carefully to his soul, so it will not perish at the time of death” (Thomas 2021, 37). Advance care planning is certainly valuable, and we completed an advance directive with Robert; however, as Thomas notes, these are secondary considerations after spiritual care. Further, those at the bedside ought not provide “false consolation” from unrealistic prognostication. With a new diagnosis of metastatic prostate cancer in his 80s, death may not be imminent for Robert; nonetheless, the time was right to spiritually prepare for death and confront his interior trial.

“The devil tempts the sick person to despair,” reminding him of prior sins, injuries inflicted on others, and failing to love God above all things, all culminating in assaulting the sick person with the words of Cain, who murdered his brother Abel: “My sin is greater than the forgiveness I deserve” (Genesis 4:13). Ironically, the devil is more morally strict than God by twisting the word of God against souls: “if you wish to enter into life, keep the commandments” (Matthew 19:17). Despair “is to be avoided above all evils as an offense against God’s mercy, which alone saves us, as the Prophet [Jeremiah] testifies: ‘The mercies of the Lord, that we are not consumed’ (Lam. 3:22). As Gregory [the Great] says, ‘Anyone who despairs of true pardon has fallen into sin; he loses mercy entirely’” (Thomas 2021, 51–52).

Against despair, the good angel provides strength in the virtue of hope in God’s mercy. “Even if you alone had committed all the sins of the world; even if you had never done penance for them or confessed them; even if you never have the opportunity to confess them in any way, still you should not despair, because in such a case inner contrition alone suffices.” The good angel exposes the devil’s deceptive and selective use of the word of God: “At whatever hour the sinner groans, he will be saved” (Ezekiel 33:12), boldly asserting “greater is the compassion of God than any iniquity,” and further encouraging the soul with countless examples of God’s boundless forgiveness from the Bible and lives of the saints (53–54). Thus, the chief weapon against despair is confident hope that God’s mercy is greater than any sin we could commit. In addition to meditating upon the good angel’s exhortation, the soul can grow in hope through the prayers and devotional practices included in the *art of dying* but especially (for Catholics) through the sacraments of penance (confession), anointing of the sick, and Eucharist.

My patient, Robert, requested someone help him pray that “God would accept him” during his hospitalization. I again offered our team chaplain to lead him through this, though he again requested that I do it, preferably during the present encounter. I acquiesced, and at the bedside I offered a prayer to this effect concluding with the Lord’s prayer, asking that God would “forgive us our trespasses” and “deliver us from evil,” invoking the confident hope of Jesus who first prayed these words. Robert thanked me with an extended handshake, a deep smile, and a tear in his eye.
